# *Chlamydomonas* Basal Bodies as Flagella Organizing Centers

**DOI:** 10.3390/cells7070079

**Published:** 2018-07-17

**Authors:** Jenna Lynne Wingfield, Karl-Ferdinand Lechtreck

**Affiliations:** Department of Cellular Biology, University of Georgia, Athens, GA 30602, USA; jlw12903@uga.edu

**Keywords:** *bld2*, *bld10*, *bld12*, centrin, striated fiber assemblin (SFA), intraflagellar transport (IFT), axoneme, central pair, microtubules, centriole

## Abstract

During ciliogenesis, centrioles convert to membrane-docked basal bodies, which initiate the formation of cilia/flagella and template the nine doublet microtubules of the flagellar axoneme. The discovery that many human diseases and developmental disorders result from defects in flagella has fueled a strong interest in the analysis of flagellar assembly. Here, we will review the structure, function, and development of basal bodies in the unicellular green alga *Chlamydomonas reinhardtii*, a widely used model for the analysis of basal bodies and flagella. Intraflagellar transport (IFT), a flagella-specific protein shuttle critical for ciliogenesis, was first described in *C. reinhardtii.* A focus of this review will be on the role of the basal bodies in organizing the IFT machinery.

## 1. Introduction

Cilia and flagella (terms referring essentially to the same cell organelles) are thread-like cell extensions. Flagella possess motile and sensory functions and are widely distributed among eukaryotes [1]. The motility of flagella serves in cell locomotion and fluid transport above epithelia. Flagellar sensing and signaling mediates, for example, cell–cell recognition in *C. reinhardtii* and the sensing of light and odor in humans. In metazoans, sensory cilia are mostly non-motile; such cilia are also referred to as primary cilia and they typically lack axonemal dyneins and other motility-related substructures. In recent years, the importance of motile and non-motile cilia for mammalian development and health has been recognized [2]. Cilia dysfunction causes a spectrum of diseases and developmental disorders referred to as ciliopathies including male infertility, situs anomalies, blindness, obesity, and cancer.

**Figure 1:**
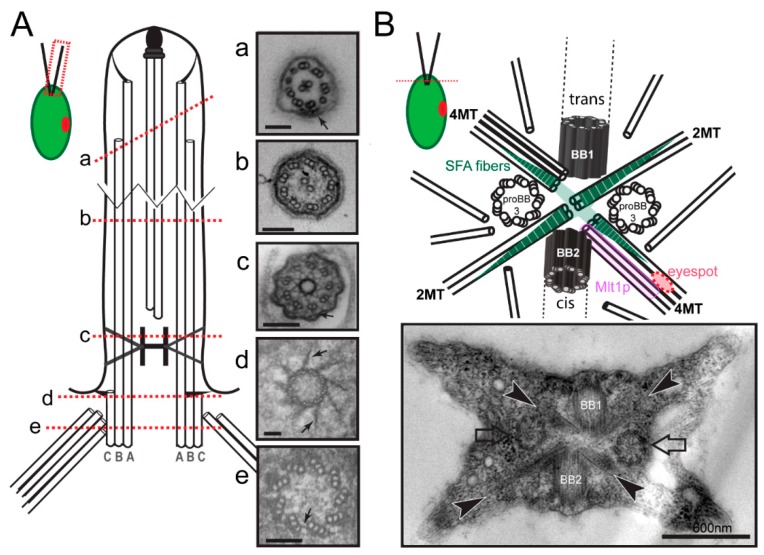
Structure of *C. reinhardtii* flagella and flagellar basal apparatus. (**A**) Cell overview. The dashed red box indicates the region shown in detail. Schematic drawing of a longitudinal section of a flagellum; the A-, B-, and C-tubules of the basal body are indicated. Red-dashed lines indicate the planes of the TEM cross-sections (**a**–**e**). (**a**) Flagellar tip with a mixture of singlet and doublet microtubules indicative for an oblique section. Arrow points to an IFT train. (**b**) 9 + 2 axoneme surrounded by the plasma membrane and glycocalyx. (**c**) Transition zone (TZ) with stellate structure, which appears as an H-shaped structure in longitudinal sections (see Figure 3B). The arrow marks a Y-linker. (**d**) The distal end of the basal body with the transitional fibers (arrows) connecting the triplets to the plasma membrane. (**e**) Triplet microtubules of the basal body. Arrow: A-C linker. Scale bars = 100 nm. (**B**) Cell overview, the red dashed line designates the plane of the cross-section. Top: Drawing of the flagellar basal apparatus with the flagella-bearing mother (BB1) and daughter (BB2) basal bodies, the probasal bodies (proBB3) and the microtubular roots consisting of 2 (2MT) or 4 microtubules (4MT). Green striated triangles: SFA fibers. The four fibers are interconnected as indicated by the green shaded area. The 4-stranded root of the daughter basal body connects to the eyespot; it is associated with Mlt1 protein, which participates in eyespot positioning. Bottom: TEM section of the flagellar basal apparatus. Arrows, probasal bodies; arrowheads, microtubular roots.

The principal structure of cilia and flagella is well conserved among species. The base of each flagellum is formed by a basal body consisting of a cylinder of nine triplet microtubules (Figure 1(Ae)). In 1954, Fawcett and Porter reported that centrioles and basal bodies are structurally identical and that centrioles convert into basal bodies during ciliogenesis [3]. The A- and B-tubules of the basal body triplets are continuous with the nine outer doublet microtubules of the axoneme; the two singlet central microtubules of 9 + 2 axonemes typical for most motile cilia originate within the cilium (Figure 1(Ab)). The region distal to the end of the C-tubules of the basal body triplets is the transition zone (TZ), which contains linkers between the microtubules and the plasma membrane and apparently functions as a ciliary gate regulating protein entry into the organelle (Figure 1(Ac)) [4]. In motile cilia, the distal boundary of the transition zone is marked by the start of the two central pair microtubules. At the distal end of flagella, the A-tubules of the peripheral doublets are longer than the B-tubules and the central pair microtubules extend even further beyond the A-tubules (Figure 1(Aa)). The central microtubules and the A-tubules are capped and linked to the flagellar membrane by capping structures of unknown composition (Figure 1(Aa)) [5]. In addition to templating the axoneme, the basal bodies function as organizing centers for the cell’s cytoskeleton. This is particularly apparent in flagellate protists such as *C. reinhardtii*, where most cell organelles including the nucleus are hardwired in position via microtubular and fibrous structures emerging from the basal bodies. In *C. reinhardtii*, the basal bodies also participate in mitosis and cytokinesis primarily by positioning the cleavage furrow with respect to the spindle apparatus [6,7]. Finally, the basal bodies organize the intraflagellar transport (IFT) machinery, a microtubule-based motility that is required for the assembly of most cilia.

**Figure 2:**
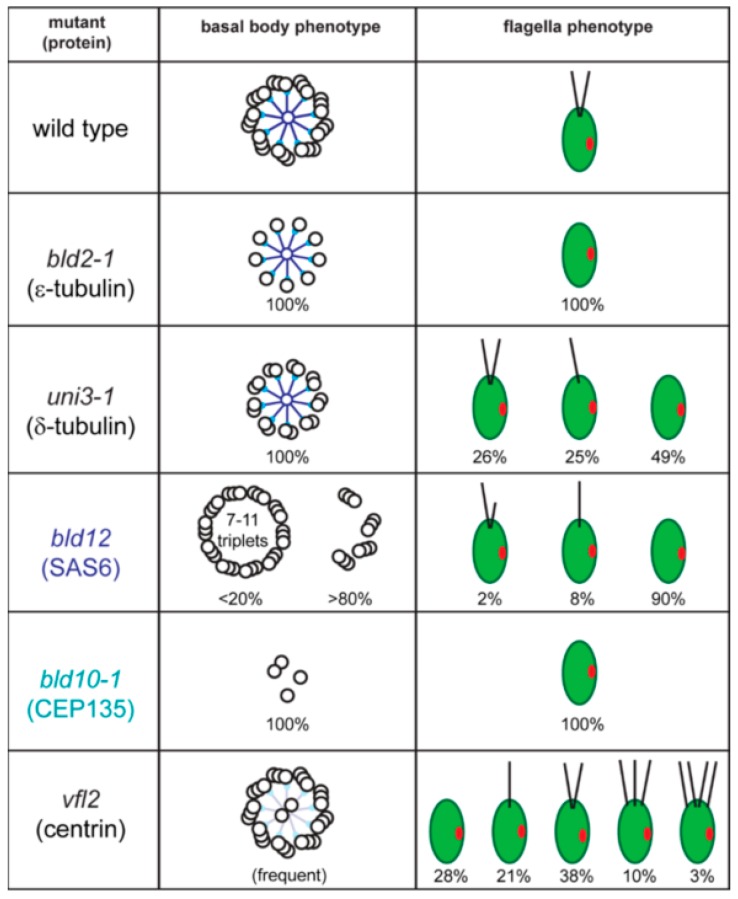
*C. reinhardtii* basal body mutants. Wild-type cells possess two flagella and the basal body consist of nine triplet microtubules and a central cartwheel. The spokes of the cartwheel consist of Bld12p/SAS6 (dark blue) and the pinheads of the cartwheel are composed of Bld10p/CEP135 (turquoise). In *bld2-1*, mutants of ε-tubulin, basal bodies consist of a ring of singlet microtubules and flagella are absent. *uni3-1* cells, mutant for δ-tubulin, build a basal body consisting of nine doublet microtubules, and possess 0, 1, or 2 flagella. *bld12* cells, mutant for SAS6, possess basal bodies with 8–11 triplets and other defects and mostly lack flagella. A portion of the *bld12* cells will build a single flagellum or a pair of unequal length flagella. *bld10-1* cells, mutant for Cep135, essentially lack basal bodies and flagella. Centrin RNAi and *vfl2* cells lack the stellate structure of the TZ causing the central pair to enter the basal body; they also have defects in basal body duplication and segregation. Values for the distribution of cells with 0–4 flagella are based on Kuchka and Jarvik, 1982.

Numerous model organisms are utilized to study the cell biology of cilia and to elucidate the molecular basis of cilia-related disease. Here, we will focus on *C. reinhardtii*, a unicellular green alga, which facilitates the biochemical, genetic, and structural analyses of basal bodies and flagella [8]. The major microtubule organizing center (MTOC) of *C. reinhardtii* during interphase is the flagellar basal apparatus consisting of two flagella-bearing basal bodies, two nascent basal bodies, and a variety of fibrous and microtubular structures emerging from the basal bodies (Figure 1B). Basal bodies with the associated structures can be isolated from *C. reinhardtii* for biochemical and proteomic analyses, and isolated basal bodies nucleate microtubules in vitro [9,10,11]. In the laboratory, neither flagella nor basal bodies are essential for *C. reinhardtii,* and numerous mutants with defects in basal body structure, number, position and function have been identified (Figure 2). Here, we will review the structure and composition of the flagellar basal apparatus of *C. reinhardtii*, analyze the development of basal bodies in *C. reinhardtii* in comparison to other systems, and describe the role of the basal bodies as the cell’s flagella organizing centers.

## 2. Basal Body Structure and Composition in *C. reinhardtii*

### 2.1. Basal Bodies

The assembly of the basal body microtubular cylinder requires γ-tubulin as indicated by knock-down experiments in *Paramecium tetraurelia* and *Trypanosoma brucei* [12,13]. In *C. reinhardtii*, antibodies to γ-tubulin decorate the wall of the basal bodies and the TZ [14]. In the latter, γ-tubulin could serve to nucleate the central pair (CP) microtubules of the 9 + 2 axoneme since in *T. brucei,* the CP microtubules are quickly lost upon γ-tubulin depletion [12]. Structures putatively corresponding to the γ-tubulin ring complex have been observed to cap the proximal end of the A-tubules in mammalian cells [15]. The assembly of the centriolar B- and C-tubule requires δ- and ε-tubulin, additional members of the tubulin family. The molecular characterization of the *C. reinhardtii uni3* mutant led to the discovery of δ-tubulin, which is encoded in the genomes of most organisms with the ability to assemble basal bodies and flagella [16,17,18]. The *uni3* basal bodies lack the C-tubules and the cells possess 0, 1, or 2 flagella (Figure 2). When a single flagellum is present, it is attached to the mother basal body (referred to here as No. 1 basal body according to the nomenclature suggested by Beech et al. 1991). In contrast, many of the daughter basal bodies (i.e., No. 2 basal bodies, which carry a flagellum in wild type) fail to assemble a flagellum in *uni3* cells, indicating that the ability of the No. 2 basal body to organize a flagellum is delayed in the absence of δ-tubulin [17]. Flagella are entirely missing in *bld2* (pronounced bald2) mutants and the basal bodies/centrioles consist of singlet microtubules [19]. *BLD2* encodes ε-tubulin, which, similar to δ-tubulin, is present in most phylogenetic groups with the ability to assemble flagella including mammals [20,21,22]. Thus, δ and ε-tubulins are involved in the assembly or maintenance of the incomplete B- and C-tubules of basal bodies and centrioles [23,24]. Antibodies to ε-tubulin decorate ring-shaped zones surrounding the basal bodies but the precise localization of these tubulin family members remains to be determined [20]. The main components of the basal bodies are α- and β-tubulin, which in green algae are acetylated and polyglutamylated with long poly-E side chains [25].

### 2.2. The Basal Body Scaffold

Basal bodies have a very complex ultrastructure with various linkers and ridges associated to the triplets in a pattern that changes repeatedly along their length. The composition and function of many of these structures remain unknown but great progress has been made in recent years. Inside the proximal portion of the basal body resides the multilayered cartwheel. The cartwheel is more prominent (i.e., longer) during basal body development and persists in mature basal bodies. The key component of the cartwheel is SAS6, which was originally identified in *Caenorhabditis elegans,* where the protein is required for centriole assembly [26,27]. SAS6 is absent in the *C. reinhardtii bld12* mutant, which assembles basal bodies with seven to eleven microtubular triplets in addition to other defects (Figure 2) [28]. In vitro, *Chlamydomonas* SAS6 homodimers self-assemble into nine-fold symmetrical rings with the extended coiled-coiled rods radiating outwards forming the nine spokes of the cartwheel [29]. More recently, the rings were shown to stack on top of each other resembling the native structure of a multi-tiered cartwheel [30]. The data suggest a key role of SAS6 for establishing or supporting the nine-fold symmetry of basal bodies and centrioles [31]. In vivo experiments using altered SAS6 suggest that the assembly of cartwheel and of the centriolar microtubules influence each other to promote the formation of the nine-fold symmetrical cylinder [32].

Bld10p/Cep135 is located in the pinheads, at the end of the SAS6 spokes of the cartwheel; the protein is critical for the nine-fold symmetry of basal bodies [33]. In contrast to *bld12*, *bld10* mutants lack intact basal bodies and flagella are absent (Figure 2) [33,34]. Bld10p/Cep135 binds the A-tubules with its N-terminal domain and SAS6 with its C-terminal region bridging the cartwheel spoke heads to the triplets [33].

Proteomic analyses of preparations enriched in *C. reinhardtii* basal bodies identified 195 proteins, including many known centriolar proteins [11]. As a validation, a subset of the human orthologues were tagged and observed to localize to the centrosome of cultures cells [11]. Here we will briefly summarize the data on two proteome of the centriole (POC) proteins. In humans, POC5 was shown to be a centrin-binding protein required for assembly of full-length centrioles [35]. POC1 is a candidate component for the A–C linkers between neighboring triplets (Figure 1(Ae)). In *Tetrahymena thermophila*, *poc1* null mutants have basal body defects ranging from missing triplets to frayed proximal ends indicating a role in basal body stability [36,37]. In *C. reinhardtii*, POC1 localizes to the mother and daughter centioles and to the doublet microtubules [38]. The protein is present in the basal body regions of *uni3-1*, *bld2*, and *bld10-1* cells, which possess damaged basal bodies, suggesting that POC1 recruitment occurs prior to and/or independently of basal body assembly.

## 3. Basal Body-Associated Structures

### 3.1. Microtubular Roots

The flagellar basal apparatus of *C. reinhardtii* organizes two distinct populations of microtubules: the 12 static root microtubules with stereotyped positions and a variable number of dynamic secondary microtubules. Each mature basal body of *C. reinhardtii* organizes two microtubular bundles or roots, one consisting of two and one consisting of four microtubules (Figure 1B) [39]. The four microtubular roots are in an X-shaped arrangement; they are highly acetylated and, as far as known, stable. Numerous additional microtubules originate near the basal bodies; these secondary cytoplasmic microtubules largely lack acetylation and are highly dynamic [40,41]. Both the microtubular roots and secondary microtubules run mostly below the plasma membrane toward the posterior end of the cell. The four microtubular roots are also key organizers of the internal cell structure. The four-stranded bundle attached to the younger basal body, for example, contacts the eyespot. Remarkably, Mlt1p (“multi1”), a protein that regulates eyespot development, is found exclusively on the 4-stranded bundle attached to the No. 2 daughter basal body (Figure 1B) [42]. In *mtl1* mutants, one or more eyespots form in association with both 4-stranded roots [43]. Positioning of the eyespot with respect to the basal bodies (and thereby the plane of the flagellar beat) is critical for the phototactic orientation of the cells, and *mlt1* mutants are non-phototactic. Thus, the microtubular roots are biochemically distinct and perform specialized functions in maintaining the cell’s inner organization and in intracellular transport. Compared to the more flexible microtubular cytoskeleton of mammalian cells, the microtubular cytoskeletons of *C. reinhardtii* and many other flagellated protists display a largely fixed architecture ensuring the correct placement of the basal bodies relative to each other and with respect to other cell organelles. One reason for this stereotyped cell architecture could be the need for a spatiotemporal coordination of cell and organelle division since many protists possess only a single copy of many organelles (e.g., plastid, microbody).

### 3.2. Fibrous Roots

The basal bodies are connected to each other, the microtubular roots, and other cell organelles by a variety of fibers, only a few of which have been biochemically characterized [44]. Together this highly organized pericentriolar material ensures that microtubules emerge from the MTOC in the right number and orientation and that the position of the cell organelles is fixed with respect to the basal bodies [45]. Two main classes of basal body-associated fibers have been characterized in detail; the contractile centrin fibers and the non-contractile striated fiber assemblin (SFA) fibers [46,47,48].

#### 3.2.1. Centrin-Based Structures

Centrin is an essential 20 kD EF-hand calcium-binding protein similar to calmodulin but possessing a helical N-terminal extension, which is critical for its capacity to self-assemble [49]. Just as with calmodulin, centrin is a calcium sensor, which changes its conformation in response to calcium binding. It is involved in a variety of processes ranging from nucleotide excision repair to the regulation of dynein arm activity [50]. Centrin is ubiquitously found in centrosomal structures, including those of organisms which lack basal bodies and centrioles, such as budding yeast, which replicates its spindle pole body using the centrin homologue CDC31 [51]. The assembly of centrin fibers often involves Sfi1p, which possesses multiple centrin-binding domains [51,52]. Centrin-decorated Sfi1p fibers are thought to resemble a beaded string, which at elevated calcium concentrations will coil resulting in the shortening of the fibers [53]. Centrin was first isolated from purified rhizoplasts, massive linkers between the basal bodies and the cell nucleus present in some prasinophytes (a subgroup of green algae), which were already observed by early light microscopists more than a 100 years ago [54].

**Figure 3:**
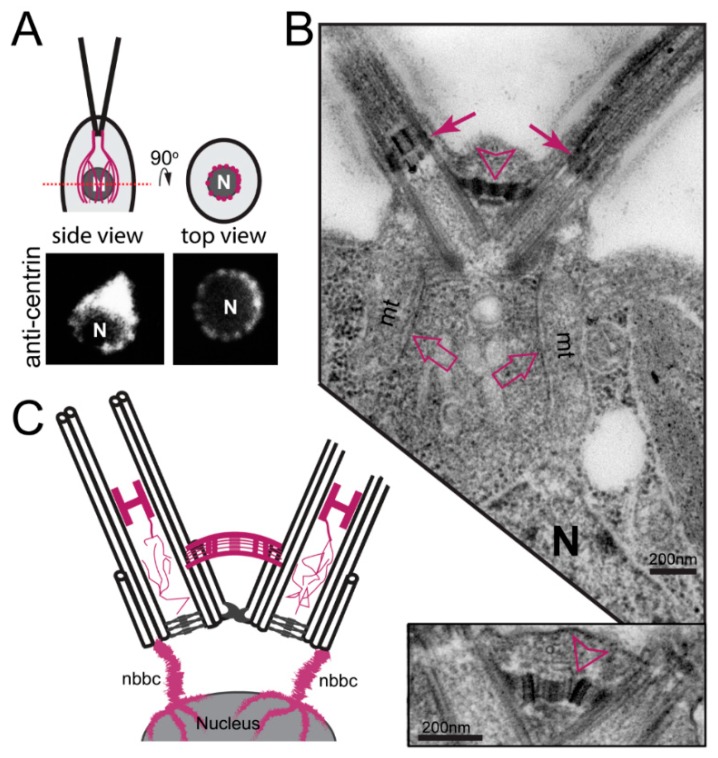
Distribution of centrin in *C. reinhardtii.* (**A**) top: Schematic presentation of cells with the nucleus and the centrin-based nucleus-basal body connectors (NBBCs) (mangenta). Bottom: anti-centrin staining in top (right) and side (left) view. The top view is a focal plane at the level of the nucleus showing the branches of the NBBCs. (**B**) An electron micrograph (EM) showing a longitudinal-section of the flagella, transition zones (closed arrows), and basal bodies. Open arrows point to the NBBCs. N, nucleus; open arrowhead, distal connecting fiber. The insert (bottom) shows the distal connecting fiber at higher magnification. (**C**) Cartoon of the centrin-based cytoskeleton (in magenta) of *C. reinhardtii*. See reference [58] for an outstanding analysis of the distribution of centrin in *C. reinhardtii* at the ultrastructural level.

In *C. reinhardtii*, centrin, originally named caltractin, was identified during a search for calmodulin-like calcium-binding proteins based on the calcium-induced shift in its electrophoretic mobility [55,56]. Centrin is present in the nucleus-basal body connectors (NBBCs) linking each basal body to the cell nucleus, the distal connecting fiber interconnecting the two mature basal bodies, and the stellate structure of the TZ linking the axonemal doublets to each other (Figure 3) [57]. Furthermore, small centrin filaments are present between the nascent and mature basal bodies and inside the basal bodies; luminal centrin fibers are early markers of the circumferential asymmetry of the probasal bodies [58]. The NBBCs of *C. reinhardtii* branch out and encage the nucleus (Figure 3A,C). Calcium-induced contraction of the NBBCs pulls the nucleus closer to the basal bodies [59]. It has been speculated that this contraction contributes to the activation of genes encoding flagella proteins after flagellar loss [60]. The NBBCs also contract premitotically reducing the distance between the basal bodies and the nucleus; they persist through mitosis and connect the basal bodies to the spindle poles [61]. Contraction of the distal connection fiber reduces the angle between the two basal bodies as it occurs when cells switch from normal forward swimming using a breaststroke-like flagellar motion to phobic backward swimming at high calcium concentrations with an undulating flagella beat pattern [62,63]. Centrin is also present in the TZ (see below) and is associated with some inner-arm dyneins [64].

In *C. reinhardtii,* centrin is encoded by a single copy gene; null mutants have not been identified and centrin/CDC31 is an essential protein in yeast. The *variable flagellar number2* (*vfl2*) mutant possesses a point mutation in centrin resulting in the loss or damage of the centrin-based fibers causing defects in basal body positioning and segregation [65,66]. The unequal distribution of basal bodies during cell division explains the phenotype, with some cells possessing extra basal bodies while other have reduced numbers of basal bodies or lack them entirely [67]. In the latter, cells apparently use de novo basal body synthesis to regain basal bodies instead of the normal pathway of templating new basal bodies next to preexisting ones [68]. Knock-down of centrin using vector-based RNAi reduced the overall number of basal bodies and flagella in addition to segregation defects, indicating that centrin fibers also participate in basal body duplication (Figure 2) [69]. Centrin RNAi cells are frequently multinucleated, indicative of a lack of spatial coordination between mitosis and cytokinesis or failed cytokinesis [69]. In centrin mutants and RNAi cells, the centrin fibers of the TZ are missing and the central pair microtubules enter the basal body causing a 9 triplets + 2 arrangement (Figure 2) [69,70]. Thus, the centrin fibers in the TZ prevent the central pair from entering the basal bodies.

#### 3.2.2. Striated Fiber Assemblin (SFA) Fibers

The four microtubular roots of *C. reinhardtii* are associated with narrowly striated fibers (~30 nm axial repeat), which are particular prominent along the 2-stranded roots (Figure 1B) [71,72]. Isolation of the striated fibers from the *Chlamydomonas* relative *Spermatozopsis similis* followed by in vitro reconstitution identified striated fiber assemblin (SFA), a ~30-kD protein, as the main component of the striated fibers [71,73,74]. In contrast to centrin, the occurrence of SFA appears to be limited to green algae, ciliates, apicomplexans including the malaria parasite *Plasmodium falciparum*, and Stramenopiles including *Phytophthora* and *Giardia* [75,76,77,78,79]. In green algae, SFA is encoded by a single copy gene, whereas multiple copies are present in apicomplexans, ciliates, and *Giardia*. Although the overall sequence conservation is low, SFAs are characterized by a central α-helical domain of ~240 residues, which is predicted to form a segmented coiled-coil based on a 29-residue repeat [78]. Parallel SFA dimers organize into 2-nm protofilaments by overlapping with their N- and C-terminal regions [80]. In *C. reimnhardtii*, the striated fibers disassemble during mitosis via phosphorylation of SFA and extended fibers redevelop during telophase [71,81].

SFA fibers typically emerge from centrosomal structures and run alongside the radiating microtubules. It is therefore widely assumed that SFA fibers serve as stabilizing or architectural elements. Based on the observations that γ-tubulin appears to be present at the SFA fibers, a role of these fibers in microtubule nucleation and the initiation/development of new basal bodies are discussed [82,83]. RNAi-based knock-down of SFA in *C. reinhardtii* results in the development of shorter than normal flagella and it has been speculated that changes in the flagellar root system could disturb the delivery of flagellar precursors to the basal bodies [84]. Functional studies in *Toxoplasma gondii* revealed that TgSFA2 and TgSFA3 are required for the development of the MTOCs of the daughter cells; loss of either protein blocks cytokinesis resulting in multinucleated cells [75]. Mutations in *T. thermophila* DisA-1, a distant relative of SFA, shortens the striated kinetodesmal fibers disrupting the alignment of the basal bodies into cortical rows [85]. Taken together, SFA-based cytoskeletal elements could function in centrosome positioning and basal body alignment.

## 4. The Basal Bodies in Mitosis

### 4.1. Cell Cycle and Mitosis of C. reinhardtii and Other Green Algae

*C. reinhardtii* has a multiple fission cell cycle with cells increasing in size during the light phase, committing to entering the cell cycle above a certain size, followed by repeated nuclear and cell divisions (1–6x) with a shortened cell cycle during the subsequent dark phase resulting in up to 64 small progeny cells [86]. Flagella are resorbed prior to entering the first mitosis and new flagella are only developed after the last cytokinesis. The daughter cells develop within the sturdy cell wall (i.e., the “chlamys”, Greek: cloak or coat) of the mother cell. After the final cell division, flagella emerge and promote the lysis of the mother cell wall to release daughter cells from the sporangium. Proteases on ectosomes, small vesicles released from the tip of the flagella, aid in the digestion of the mother cell wall [87]. Thus, *C. reinhardtii* mutants lacking flagellar motility, and especially those lacking flagella entirely, often remain enclosed in the mother cell wall, resulting in palmelloid growth.

*C. reinhardtii* has a semi-open mitosis with large fenestrae at the spindle poles [88]. Following resorption of the flagella in early mitosis, the two pairs of basal bodies separate. In *C. reinhardtii*, the basal bodies duplicate in late prophase/early metaphase and four basal bodies are present near each spindle pole [6]. An amorphous ring is present prior to the formation of the cartwheel and missing from mature basal bodies suggesting a function as a transient scaffold during basal body assembly [16]. Cep70 is a putative component of this ring and is primarily present in pro-basal bodies, and largely absent from the mature flagella-bearing basal bodies [89]. Further, knock-down of Cep70 in *C. reinhardtii* interferes with the recruitment of Bld12p/SAS6, ε-tubulin and Bld10p/CEP135 to the sites of basal body assembly and impairs ciliogenesis [89]. New basal bodies are first observed orthogonal to their mothers but they disengage early and continue to develop between the microtubular roots with their proximal ends close to the SFA fibers (Figure 1B) [6]. As in mammalian cells, the A-tubule of the triplets are initially longer and the B- and C-tubules are added laterally onto the A-tubules; as the new basal bodies enter the dormant state, the overhanging A-tubules are trimmed and the probasal bodies shorten [6,15]. This and other observations have fueled speculations that minus-ends polymerization of the triplet microtubules could contribute to basal body growth [83,90].

The four-stranded microtubular roots, one from each set of basal bodies, form an overlapping antiparallel bundle known as the metaphase band, which persists throughout mitosis and arcs over the nucleus [91,92]. The microtubular roots apparently ensure the correct positioning of the mitotic spindle and cleavage furrow and, in strains lacking the root microtubules, coordination between the two frequently fails [7]. The basal bodies remain somewhat offset from the spindle poles, and the formerly flagella-bearing basal bodies remain attached to the plasma membrane throughout mitosis [6].

Evidence from other green algae suggests that the basal bodies, while not directly positioned at the spindle poles, could have a role in initiating spindle pole development. In *Dunaliella bioculata* and *S. similis*, the daughter basal bodies touch the nuclear envelope during prophase and spindle assembly will then commence at these contact sites [61,83]. Contact between the basal bodies and the nucleus is facilitated by the premitotic contraction of the NBBCs [60]. In *S. similis*, a multilayered cartwheel protrudes from the proximal end of the daughter basal bodies and contacts the nuclear envelope; singlet microtubules in various orientations originate on the protruding cartwheel indicative for MTOC activity [83]. A cartwheel with 8 or more layers has been also observed in *C. reinhardtii* [93]. In contrast to *C. reinhardtii*, many green algae lacking a solid cell wall divide while maintaining their flagella. In *Dunaliella*, for example, the basal bodies remain at the cell’s apex and are connected to the more posterior spindle poles via centrin fibers. Earlier, a layered spindle pole body-like structure is present between the daughter basal bodies and the nuclear envelope of *D. bioculata* [61]. Green algal cytokinesis involves a phycoplast, in which microtubules are arranged parallel to the developing cleavage furrow.

### 4.2. The Basal Body and Flagellar Developmental Cycle

**Figure 4:**
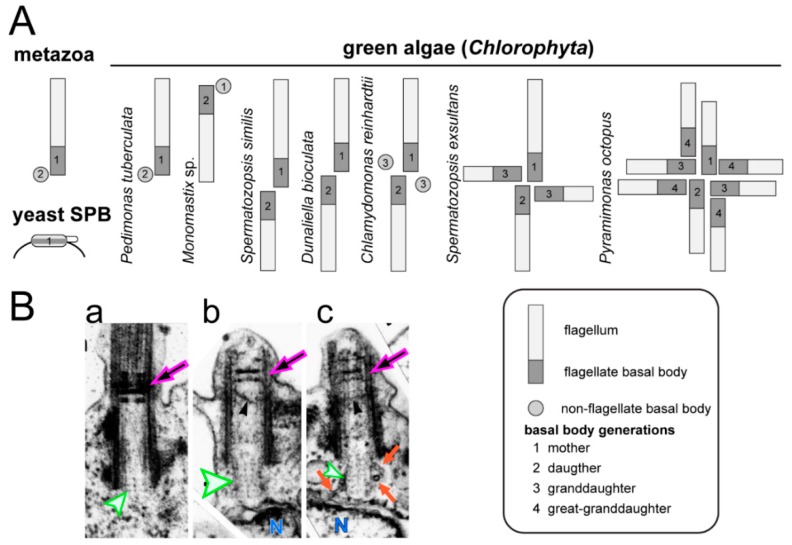
Basal body number and maturation in green algae. (**A**) The mammalian centrosome consists of one mother centriole (1), which might bear a primary cilium, and a daughter basal body (2); a new generation of basal bodies (3) is formed in S phase. Saccharomyces cerevisiae possess a single spindle pole body (SPB) corresponding to one generation of a centrosomal organizer. By comparison green algal centrosomes are more variable with respect to the number of basal bodies, flagella development. (**B**) TEM images of basal bodies of the green alga *S. similis*. In mature basal bodies (**a**), the cartwheel typically consists of three tiers embedded into the axonemal cylinder (green arrowhead). In premitotic cells, the No. 2 basal bodies (**b**,**c**) dock to the plasma membrane and develop a protruding cartwheel of ~8 tiers that touches the nuclear envelope (N). Microtubules radiate from the protruding cartwheel suggesting a microtubule organizing center (MTOC) activity. Note the developing TZ. Reprinted with permission from Lechtreck and Grunow (1999) [83].

Metazoans typically possess a pair of centrioles referred to as the mother and the daughter centriole, representing two generations of centrioles (Figure 4A). The mother centriole can possess a primary cilium in G1; during S-phase, the primary cilium is resorbed, basal bodies duplicate and the basal body pairs disengage. Thus, the development of a centriole/basal body from its inception in S-phase to the development of a primary cilium typically requires more than one cell cycle and two mitoses.

Compared to most metazoans, green algae display great variability with respect to basal body number and the timing of their maturation (Figure 4). One flagella-bearing and one non-flagellated basal body are present in the green alga *Pedimonas tuberculate* [93]. In this species, it is the older mother basal body that carries the flagellum while in *Monomastix* sp., the flagellum appears to be attached to the younger basal body. In the latter case, the flagellum is resorbed as the No. 2 basal body matures into the No. 1 mother basal body. In *C. reinhardtii*, basal bodies are assembled during early mitosis, spend one cell cycle as dormant No. 3 probasal bodies, another cell cycle as flagella-bearing No. 2 basal bodies connected to the eyespot, before reaching the final developmental state and position of the No. 1 basal body. Thus, *C. reinhardtii* possesses three generations of basal bodies during interphase. The prasinophyte green alga *Pyramimonas octopus* possess eight flagella-bearing basal bodies corresponding to four generations; the maturation time from the assembly of a basal body to reaching its final state and position requires more than four cell cycles [94]. Of note, all these basal bodies are centrosomal basal bodies. They possess structural markers allowing for identification of each generation, assemble at a specific stage of the cell cycle, are connected to the poles of the spindle during mitosis and are inherited in a semi-conservative fashion during cell division. These features set green algal basal bodies apart from the non-centrosomal cortical basal bodies of ciliates and other multiciliated cells, which are neither linked to the spindle poles during mitosis nor function within a single central MTOC.

In addition to the differences in basal body number and maturation time, variations in the timing of flagella formation and the impact of basal body age on the size of the attached flagellum occur between different green algal species [93,95]. The mother and daughter basal bodies of *C. reinhardtii* assemble flagella of the same length, but the two flagella respond differently to changes in calcium, a behavior that is important for phototactic steering [96]. Ultrastructural and biochemical differences between the two flagella of *C. reinhardtii* remain to be identified. In other green algae, the length of the flagella depends on the age of the basal body that organizes the flagellum. The green flagellate *S. similis*, for example, possesses one long and one short flagellum, which assemble on the older and younger basal body, respectively [97,98]. In its quadriflagellate relative *Spermatozopsis exsultans*, short flagella are present on the two No. 3 basal bodies, a medium length flagellum exists on the No. 2 basal body, and a long flagellum is present on the No. 1 mother basal body [93]. Many algae remain flagellated during division and, as the basal bodies mature and travel to their new position within the cell, the length of the attached flagellum changes, a process referred to as the flagella developmental cycle [95]. Heterokont protists often possess ultrastructurally distinct flagella, i.e., a short smooth and a long hairy (i.e., with mastigonemes) flagellum. During cell division, the long hairy flagellum matures into a short smooth flagellum [99]. This suggests that protein entry into flagella depends on the developmental stage of the basal bodies. In summary, considerable differences exist among green algal species with respect to the number of generations of centrioles/basal bodies within a cell, their position with respect to the spindle poles and the timing of basal body duplication and maturation. In mammalian cells, biochemical markers for the mother and daughter centrioles have been identified [100]. However, the biochemical differences between the basal bodies in green algal cells with three or four generations of the organelle, as well as the processes that govern basal body maturation, remain elusive.

## 5. Basal Bodies as Organizing Centers for Flagella

The best established function of basal bodies and centrioles is the organization of flagella. Basal bodies contribute to ciliogenesis in several ways. They function as templates for the microtubular doublets of the flagellum and recruit IFT proteins, which deliver ciliary precursors into the growing organelle. Furthermore, structures linking the basal bodies to the plasma membrane control the exchange of membrane and soluble proteins between the two compartments [101].

### 5.1. Templating of the Axoneme

The A- and B-tubules of the basal body are continuous with the outer doublet microtubules of the flagellar axoneme while the C-tubules terminate within the cell body (Figure 1A). Mutants with an abnormal number of triplets often fail to assemble an axoneme or give rise to axonemes with 8–11 doublets, formally demonstrating the critical role of the basal bodies for the nine-fold symmetry of the axoneme [28]. The axonemal doublets are densely decorated with axonemal substructures such as dynein arms and radial spokes; they also serve as tracks for the IFT motors. IFT traffic and the binding of axonemal substructures typically occur along specific protofilaments or groups of protofilaments [102] but the mechanism by which these proteins recognize their designated poitions on the doublets remains unknown. Recent high-resolution data in *T. thermophila* revealed that the protofilaments of the axonemal doublets possess different local angles, i.e., neighboring protofilaments are tilted with respect to the ideal orientation in a symmetric microtubule [103]. It is tempting to speculate that this peculiar arrangement of protofilaments establishes specific binding sites along the surface of the axonemal doublets and that this configuration is already predetermined in the basal bodies. In mammalian cells, CEP120 localizes to the distal ends of the C-tubules and promotes centriolar elongation [104,105]. In *C. reinhardtii*, a CEP120 homologue is encoded by *UNI2* and *uni2* mutants, often fail to assemble two flagella [106]. In *uni1 uni2* double mutants a subset of the triplet microtubules reaches into the TZ indicating a defect in C-tubule termination [107].

### 5.2. The Transition Zone

The region between the end of the C-tubules and the beginning of the central pair is referred to as the TZ. In comparison to the basal body/centriole and the axoneme, the ultrastructure of the TZ varies considerably between species. A hallmark of the *C. reinhardtii* TZ is the stellate structure, composed of two helices of centrin filaments that interconnect the doublets on the inside of the axonemal cylinder (Figure 1(Ac)) [16]. Calcium-induced contraction of these fibers is thought to contribute to flagellar shedding by microtubule severing at predetermined sites above the TZ, a response that reduces the exposed surface of the cell and renders cells motionless, potentially confusing predators [108,109]. Acid-induced deflagellation fails in a calmodulin expression mutant; the *cam* mutant still sheds its flagella in response to chemicals and mechanical shearing suggesting that the deflagellation machinery remains intact [110]. A potential role of katanin in severing the TZ microtubules was not confirmed since the central pair-less katanin mutants *pf15* and *pf19* deflagellate normally [111,112]. The TZ remains with the basal bodies during flagellar amputation but it dissociates from the basal body proper during premitotic flagellar resorption, and a TZ remnant enclosed in a membrane vesicle is shed from the cell [113]. Proteomic analysis of these TZ remnants identified most known TZ components of *C. reinhardtii* (such as NPHP4 and CEP290, see below) and several proteins of the endosomal sorting complexes required for transport (ESCRT), which could function in membrane sealing during flagellar and TZ shedding [114]. 

Besides its roles in flagellar shedding and the subsequent sealing of the wound, the TZ is a cellular gate privileging specific proteins for entry into the cilium and preventing the entrance of others [115]. This contributes to cilia having a specific protein composition, ensuring proper function. It is unclear whether the TZ is an active gate, where enzymatic reactions mediate protein passage comparable to the nuclear pore complex, or merely a physical barrier that delays and prevents entry of larger proteins by diffusion. Ciliary membrane proteins move laterally from the plasma membrane into the cilium, and both the Y-linkers (Figure 1(Ac)) and the ciliary necklace, a ring of intramembrane particles, are thought to be involve in the admission process [116,117]. The necklace and the Y-linkers are widely conserved elements of the TZ and contain a conserved set of proteins including CEP290 and nephrocystin4 (NPHP4) [118,119]. In humans, mutations in NPHP4 cause nephronophthisis, a sever kidney disease affecting children, and CEP290 mutations cause a range of conditions including kidney anomalies, blindness and brain malformations. *C. reinhardtii nphp4* and *cep290* mutants harbor biochemical defects in their flagella including both abnormal accumulation and loss of proteins, confirming the role of the TZ in establishing and maintaining the cilia’s protein content [118,119].

The flagellar central pair (CP) originates above the TZ and γ-tubulin is located in the TZ suggesting a role in the nucleation of the CP microtubules [14]. However, when CP assembly is initiated in full-length CP-deficient flagella, CP markers first become apparent subdistally to the flagellar tip [120]. Also, mutants lacking the radial spokes or with extra-wide axonemes with more than nine outer doublets often develop two CPs, each consisting of two microtubules and the CP projections [120,121,122]. This suggests that the number of CP microtubules might be regulated by the space available inside of the axonemal cylinder rather than by a defined nucleation site near the base.

### 5.3. Recruitment of Intraflagellar Transport (IFT) Proteins and IFT Train Assembly

The plus-ends of the axonemal microtubules point away from the cell body. Thus, tubulin and other axonemal proteins are added to the distal end of the axoneme during flagellar growth [123,124]. Flagella are devoid of ribosomes, and flagellar proteins are postranslationally moved into the organelle. Moreover, proteomic studies revealed the presence of 600–1000 distinct proteins in cilia, many of which are highly enriched inside the organelle compared to their concentration in the cell body [125]. Thus, it has been long suspected that flagellar assembly involves motor-based transport of proteins from the cell body into the cilia [124]. Indeed, flagellar assembly depends on intraflagellar transport (IFT), a protein shuttle dedicated to flagella that selects proteins in the cell body, transfers them across the TZ, and unloads them in the flagellum mostly near the tip. Here, we will focus on the role of the basal bodies as organizers for IFT; numerous recent reviews on IFT are available [126,127].

**Figure 5:**
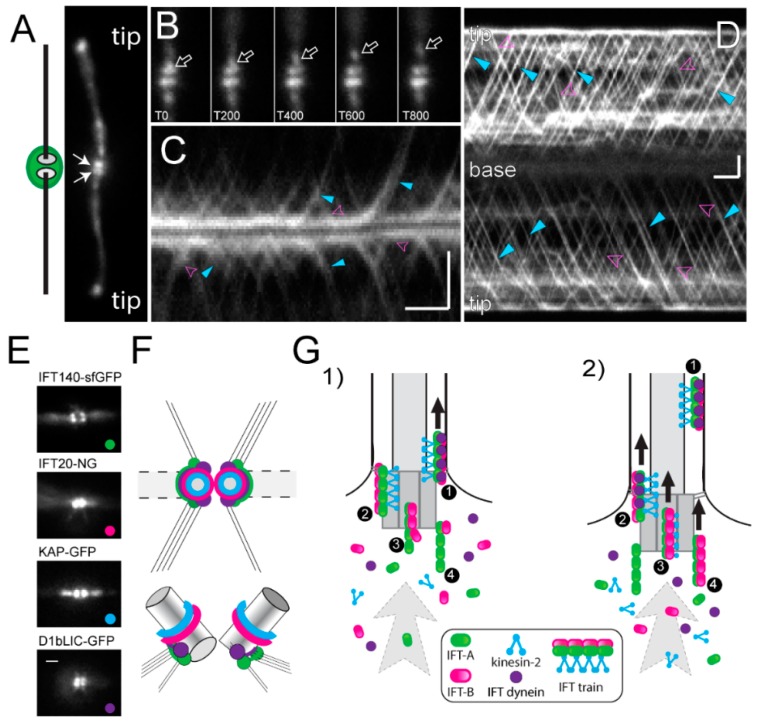
Basal bodies as organizer for IFT. (**A**) Overview and total internal reflection fluorescence (TIRF) image of a live cell expressing mNeonGreen-IFT54. The basal body pools (arrows) and the flagellar tips are marked. (**B**) Series of still images depicting the departure of an anterograde IFT train (arrow) from the basal body pool. T indicates the time in ms. (**C**) Kymogram (plot of time vs. position) showing the departure and arrival of IFT trains at the flagellar base. Turquoise arrowheads, anterograde trains; pink arrowheads, retrograde trains. Note changes in the signal strength of the basal body pools as trains exit or arrive. (**D**) Kymogram of IFT traffic in the two flagella. The flagellar base and tip, anterograde trains (turquoise) and retrograde trains (pink) are indicated. Bars (**C**,**D**) = 2 μm 2 s. (**E**) Still images of live cells expressing IFT140-sfGFP (IFT-A; green), NG-IFT20 (IFT-B, red), KAP-GFP (anterograde motor; turquoise) and D1bLIC-GFP (retrograde motor; purple), each of which pool near the two flagella-bearing basal bodies. 10-frame average images are shown for clarity. Bar = 1 μm. (**F**) Schematic presentation of the distribution of IFT proteins shown in E in the pool in top and side view based on focal series. (**G**) Model of IFT train assembly. IFT precursor complexes are recruited from the large cell body pool to the basal bodies and assembled into trains by sequential addition of the distinct subcomplexes; complete trains will enter the flagellum. Panels **B**–**D** are reprinted in modified form Wingfield et al. 2017 [148].

The IFT machinery consists of the IFT motors and the IFT particle proteins, which together assemble into repetitive multi-megadalton arrays (IFT trains) that traffic up and down the axonemal doublet microtubules. The IFT motors are a heterotrimeric kinesin-2 for anterograde transport to the flagellar tip and IFT dynein for retrograde transport back to the cell body [128,129]. The biochemically stable IFT particles consists of 22 distinct IFT proteins, organized into three subcomplexes (IFT-A composed of IFT43, IFT121, IFT122, IFT139, IFT140 and IFT144, IFT-B1 consisting of IFT22, IFT25, IFT27, IFT46, IFT54, IFT56, IFT70, IFT74, IFT81 and IFT88, and IFT-B2 with the IFT20, IFT38, IFT54, IFT57, IFT80 and IFT172 subunits) [127,130]. The IFT trains inside cilia possess a periodic substructure and each repeat is thought to consist of one or two IFT particles [128,131]. Anterograde trains are ~200–300 nm in length; retrograde trains are about the same length but less structured. Anterograde trains tend to stall inside cilia (at least inside cilia attached to glass surfaces as they are typically used for imaging experiments) and then assume an extended configuration with a length of ~700 nm [102,132,133,134]. More than 90% of the total amount of the IFT proteins are present in the cell body with a portion pooling near each basal body; the IFT trains emerge from this basal body-associated pool of IFT proteins (Figure 5A–D) [135,136,137]. In contrast to the discrete structure of ciliary IFT trains, the organization of the pool of IFT proteins surrounding the basal bodies remains elusive and bona fide IFT trains are only seen near the distal end of basal bodies, i.e., near the transitional fibers (Figure 1(Ad)) [138]. This suggests that the basal bodies organize the IFT proteins into trains.

Protein interactions within the 22-subunit IFT particle and its subcomplexes have been intensively studied in vitro [139,140,141,142]. Furthermore, the hierarchy by which IFT proteins are recruited to the basal body is emerging based on the analyses of IFT and basal body mutants, many of which lack flagella [137]. IFT52, for example, is required for the accumulation of IFT46 at the flagellar base [143]. IFT-A proteins (e.g., IFT139) accumulate at the base of *ift74-1* mutants but their entry into the flagellum is diminished [144]. In the IFT dynein mutant *d1blic*, IFT particle proteins still enter cilia, suggesting that the retrograde motor is not required for train assembly [145,146]. The core IFT-B1 protein IFT52 associates to the transitional fibers of the basal bodies (Figure 1(Ad)) [136]. *bld-2* cells lack the transitional fibers (Figure 1(Ad)) and the IFT motor kinesin-2 fails to accumulate near the basal bodies suggesting a critical role of the transitional fibers for motor recruitment [137]. Recently, it was shown that IFT80 dimerizes, which could underlie the formation of IFT particle dimers [147]. However, how the higher-order IFT trains are assembled near the basal bodies is only emerging.

Using fluorescence recovery after photobleaching (FRAP) and fluorescence loss in photobleaching (FLIP) analyses, we recently showed that the IFT system of *C. reinhardtii* is essentially open, meaning that IFT proteins are recruited from the large soluble cell body pool to the basal body region and assembled into trains that enter the flagellum. Most IFT proteins returning from the cilia to the basal bodies via retrograde IFT are not reused directly but released into the cell body pool. After photo-bleaching of the basal body-associated pool, the traffic of fluorescent IFT trains into the attached flagellum is interrupted for a few seconds during which the bleached IFT proteins exit the pool in form of IFT trains [148]. The length of this interruption provides a measurement for the time an IFT protein needs to transition through the basal body pool from its recruitment to its release into the cilium. Interestingly, the recovery times varied between the different IFT protein complexes and the motor from ~2.5 s for KAP-GFP (a kinesin-2 subunit) to ~9 s for IFT-A proteins. This suggests that IFT train assembly commences with the recruitment of IFT-A complexes to the flagellar base, followed by the association of IFT-B2, then IFT-B1, and finally the kinesin-2 motor. In agreement with this model, kinesin-2 and the IFT-B proteins are located more distally along the basal body axis, i.e., closer to the TZ where complete functional IFT trains are expected (Figure 5E,F) [137,144,149]. Our data suggest that IFT trains in different stages of assembly line up near the basal bodies (Figure 5G) [148].

Tubulin and IFT dynein, which are cargoes of anterograde IFT trains, are recruited briefly before the trains depart. Phosphorylation of the FLA8/KIF3B subunit regulates the interaction between kinesin-2 and IFT-B, controlling the rate on IFT entry into the flagellum [150]. The entry of the trains into the flagellum is also controlled by the centrosomal proteins FOP, CEP19, and the small GTPase RABL2 [151,152]. After trains pass through the TZ they processively make their way to the tip, occasionally dropping off some cargo along the way [153]. At the tip, the trains fragment, depositing most of the axonemal cargoes, and reassemble into retrograde trains [154]. Upon returning to the flagellar base, the IFT trains disassemble and most proteins are released back into the soluble cell body pool, except for a fraction of the IFT-B proteins, which appear to be reused in subsequent anterograde IFT trains without exiting the basal body pool [148].

Does the developmental age of the basal bodies affect IFT? The two axonemes of *C. reinhardtii* flagella respond differently to calcium suggesting that they are biochemically distinct [96]. Furthermore, many protists assemble flagella of distinct length and/or ultrastructure (e.g., with and without mastigonemes) on the same cell depending on the developmental state of the associated basal body. This suggests that basal bodies participate in the processes that determine which cargoes will enter the attached cilium. By extension, changes in the quantity and quality of the proteins recruited to a basal body could regulate changes in cilia composition and size as they occur during development and signaling. Thus, the basal bodies not only template the axonemal doublets, but their activity also concentrates IFT proteins at the flagellar base, participates in IFT trains assembly, cargo selection, cargo loading, and the regulation of IFT train entry and frequency. Understanding how the basal bodies perform these roles is critical for our understanding of cilia assembly.

## Abbreviations

CP	central pair
TZ	transition zone
POC	proteome of the centriole
NPHP	nephronophthisis
GFP	green fluorescent protein
IFT	intraflagellar transport
